# Individual Differences in Moral Development: Does Intelligence Really Affect Children’s Moral Reasoning and Moral Emotions?

**DOI:** 10.3389/fpsyg.2016.01961

**Published:** 2016-12-20

**Authors:** Hanna M. Beißert, Marcus Hasselhorn

**Affiliations:** Department of Education and Human Development, German Institute for International Educational ResearchFrankfurt am Main, Germany

**Keywords:** intelligence, cognitive development, moral development, moral reasoning, moral emotions

## Abstract

This study investigates the relationship between intelligence and individual differences in children’s moral development across a range of different moral transgressions. Taking up prior research that showed morality and intelligence to be related in adolescents and adults, the current study wants to test if these findings can be extended to younger children. The study was designed to address some of the shortcomings in prior research by examining young children aged between 6 years; 4 months and 8 years; 10 months, using a broad concept of moral development including emotional aspects and applying an approach that is closely connected to children’s daily lives. Participants (*N* = 129) completed a standardized intelligence test and were presented four moral transgression stories to assess moral development. Results demonstrated that findings from prior research with adolescents or adults cannot simply be extended to younger participants. No significant correlations of moral development and intelligence were found for any of the presented stories. This provides first evidence that – at least in middle childhood – moral developmental status seems to be independent from children’s general intelligence assessed by figural inductive reasoning tests.

## Introduction

This study examines the relationship between intelligence and individual differences in children’s moral development. As common in the field of moral development research, we use the term ‘moral development’ not only to describe the course of moral development but also for the characterization of an empirically ascertainable moral developmental status. Researchers from different fields have documented numerous facets of the development of children’s morality, by describing moral judgments (e.g., Kohlberg, 1969), moral reasoning (e.g., Eisenberg et al., 1983), moral emotions (Eisenberg, 2000), or moral motivation (e.g., Nunner-Winkler, 2007). Although in general, there are clear patterns of moral development, children vary in the speed and levels of achieved moral development (Nunner-Winkler, 1998). It remains to be clarified which factors explain these individual differences in children’s moral development. Following Kohlberg’s (1969) idea of a stage model which assumes that moral stages are structured in the same developmental sequence as intelligence operations, it is self-evident to consider intelligence as a factor to adequately explain differences in moral development.

Kohlberg (1969, 1975) as well as researchers from the neo-kohlbergian approach (e.g., Rest et al., 1999) have theoretically elaborated and empirically examined the idea that cognitive and moral development are interrelated and go hand in hand. Moral development requires a variety of abilities which are related to cognitive processes and their efficiency: complex situations need to be interpreted, relevant information must be selected and processed, information needs to be integrated, perspectives need to be coordinated, consequences of actions need to be anticipated, inferences need to be made, social norms (e.g., equity, fairness, etc.) need to be restructured and applied (Rest, 1986; Dentici and Pagnin, 1992; Derryberry et al., 2005). Therefore, it seems plausible that moral judgments or decisions require reasoning abilities and problem solving skills (Derryberry et al., 2005). In other words, a well-developed morality requires a complex organism with the potential for abstract reasoning. And according to Silverman (1994, p. 112) ‘high intelligence is synonymous with abstract reasoning ability and complexity of thought,’ thus intelligence can be assumed to affect moral development. Further, most of these necessary cognitive processes are connected to information processing. Thus, information processing capacities are crucial and since higher intelligence is associated with more efficient information processing (Kail and Salthouse, 1994), more intelligent people should be better able to integrate and coordinate information efficiently and make more sophisticated moral judgments and justifications.

Empirical evidence for this was provided by research on the gifted in which giftedness was found to be related to advanced moral reasoning skills. For example, Derryberry et al. (2005) found that gifted adolescents were advanced in their moral judgments in comparison to a group of non-gifted children and in both groups, intelligence was a significant predictor for the moral scores. Likewise, Howard-Hamilton and Franks (1995) could show that the gifted high school students in their study were advanced in their moral development showing mean moral scores which are near the level of college students. Lee and Olszewski-Kubilius (2006) also showed that gifted students are more morally sensitive and advanced in moral reasoning than students from unselected samples. A range of older studies similarly indicated a higher level of moral development for gifted students (e.g., Thorndike, 1940; Karnes and Brown, 1981; Tan-Willman and Gutteridge, 1981; Janos and Robinson, 1985; Howard-Hamilton, 1994; Silverman, 1994).

However, four major issues need to be addressed concerning prior research in this field. Firstly, most of the studies examined adolescents or even young adults. Only few studies have dealt with younger children. Yet, there is some evidence that also in younger age groups gifted children are advanced in moral reasoning compared to their non-gifted peers. For instance, already Terman in his ‘Genetic Studies of Genius’ found gifted children to show an advanced moral development making moral decisions on moral levels which are usually found in late adolescence (as cited in Janos and Robinson, 1985). Likewise, Kohlberg (1964) and Gross (1993) found that highly gifted children had very advanced abilities in conceptualizing moral issues and provided moral reasoning on levels which are usually prevalent only in very few adults. Finally, Chovan and Freeman (1993) could show that the gifted children in their study achieved higher levels of moral reasoning than their peers of average ability. Regardless, more research with younger children is needed to examine the role of intelligence in periods which are crucial for moral development.

Further, one difficulty is that in most of these earlier studies, groups of gifted children were determined by their participation in certain programs for the gifted, e.g., summer camps. Participation in such programs is usually based on teacher nominations which have been shown to be problematic (Rost and Hanses, 1997; Baudson, 2010). In many studies, giftedness is not precisely defined and intelligence is in many cases not even measured at all (Rost and Czeschlik, 1994). Further research is needed which systematically measures intelligence as well as moral development, and is not merely based on samples of pre-selected students.

Moreover, most of the pertinent research has been done in the (neo)-kohlbergian tradition using either the Moral Judgment Interview (Colby and Kohlberg, 1987) or the Defining Issues Test (DIT, Rest, 1979; Rest et al., 1999). The Moral Judgment Interview consists of a series of moral dilemmas with open ended questions designed to “elicit a subject’s own construction of moral reasoning, moral frame of reference or assumptions about right and wrong and the way these beliefs and assumptions are used to make and justify moral decisions” (Colby and Kohlberg, 1987, p. 61). Its coding procedure results in a specification of the stage structure and definition of the developmental sequences of the specific moral concepts within each stage. The DIT, in contrast, is a multiple-choice instrument to measure moral judgment development with the help of different moral dilemmas asking participants to rate and rank a set of items in terms of their moral importance. The idea underlying the DIT is that reading the moral dilemmas and the issue statements activates moral schemas (limited by the extent that a person has already developed them). Both instruments have in common that in the dilemmas, different moral principles conflict each other. This is interesting in terms of moral reasoning from a rather philosophical point of view, but it is less relevant in terms of children’s observable moral development. These dilemmas do not reflect the type of moral conflicts which children usually have to deal with. In children’s daily lives, moral dilemmas rather consist of conflicts between moral obligations on the one hand and personal desires or needs on the other hand (Hoffman, 1991; Keller, 2001). Thus, when measuring children’s moral reasoning competencies, it is necessary to use realistic problems which are close to the world of children’s experiences (Keller, 2001).

Hence, there is a lack of research that investigates the relationship between moral development and intelligence using an instrument that captures the kind of moral conflicts which are common in children’s experiences of the world. It should be tested whether findings from prior research can be generalized to assessments of morality which are less abstract and closer to everyday life.

Finally, another important issue is that morality or moral development is not limited to moral cognitions, i.e., moral judgments and moral reasoning. Moral cognition is only one dimension of morality. Most developmental researchers would agree that moral development includes both – cognitive and emotional aspects (Malti et al., 2009b; Gibbs, 2013). Nevertheless, moral cognition (i.e., moral judgments and moral reasoning) and moral emotions are closely connected and in continuous interaction as the emergence of moral emotions is dependent on moral cognitions (Dentici and Pagnin, 1992; Malti and Latzko, 2010). Even though the two aspects are somehow interdependent, there seems to be some disparity in the course of development. Young children already show an elaborate moral knowledge and are also able to reason in morally adequate ways about moral transgressions (e.g., Turiel, 1983), yet they do not understand the significance of moral transgressions for their self-evaluative and empathic emotions before the age of 7 or 8 years and thus typically attribute positive emotions to hypothetical wrongdoers in moral transgressions scenarios (Nunner-Winkler and Sodian, 1988; Malti and Krettenauer, 2013). From a viewpoint of functional theories of emotions, this means that morality (i.e., moral knowledge and understanding) has not yet gained motivational force (Nunner-Winkler and Sodian, 1988). Children need to develop moral motivation first, which can be understood as the willingness to follow moral rules which a person understands to be valid, even if this entails personal costs or conflicts with one’s own interests (Nunner-Winkler, 2007, 2009). Accordingly, moral development can be understood as a two-step process: first, children develop moral knowledge which enables moral judgments and moral reasoning, and only in a second step, do they develop moral motivation based on moral emotions (Nunner-Winkler, 2007). So, these two aspects represent two dimensions of morality that are both important for moral behavior and are closely connected. Moral cognitions evoke knowledge structures enabling the emergence of moral emotions (Dentici and Pagnin, 1992). Moral emotions, in turn, engage motivational forces which are important for the development of moral behavior (Dentici and Pagnin, 1992; Hoffman, 2000; Malti and Latzko, 2010).

Hence, the measurement of morality or moral development should not be restricted to moral judgments and reasoning. Both, moral judgments and moral emotions (and the respective reasoning) should be considered to ensure that a complete picture of children’s moral development is obtained. And in terms of relations to intelligence, not only moral judgments and moral reasoning should be related to cognitive abilities. We assume that intelligence is related to moral emotions as well as to moral judgments and moral reasoning. Moral emotions are complex emotions and they differ from basic emotions insofar as they have a strong cognitive component and emotion attribution in morally relevant situations necessarily involves a substantial degree of cognitive processing (Malti et al., 2009b; Malti and Krettenauer, 2013). However, research on relations of intelligence and morality is mostly limited to measures of moral judgment or moral reasoning and there is a lack of research that investigates the role of intelligence in moral emotions. Thus, research is needed that systematically examines the relation between cognitive factors, i.e., intelligence, and different facets of moral development such as moral reasoning, moral emotion attribution and moral motivation.

The current study examines the relation between children’s moral development and intelligence across a range of different moral transgressions. The study was designed to test the hypothesis that intelligence contributes to individual differences in children’s moral development by addressing some shortcomings of prior studies.

While most prior studies have dealt with adolescents or young adults, to evaluate the role of intelligence in moral development, it is necessary to focus on individuals who are in a developmental period of large changes in the relevant behavior. In our study, we interviewed children aged between 6 and 9 as during this developmental period, children show an awareness of a range of moral principles, and research demonstrates that developmental changes in children’s understanding of moral principles occur during this period (Eisenberg et al., 1983; Smetana, 2006).

Furthermore, other measurement approaches of morality are needed which not only include moral reasoning about abstract dilemmas as in the (neo-)kohlbergian tradition. There is a lack of studies that administer moral variables in a less abstract way, i.e., closer to children’s actual moral behavior in everyday life. The current study aims at closing the gap by measuring children’s morality using an approach which better mirrors the type of moral challenges children face in their daily lives. By using moral transgression stories, the current study applies an instrument that is capable of capturing different facets of moral development such as moral judgments and moral reasoning as well as moral emotions and moral motivation. Furthermore, four different stories were used to measure children’s morality across a range of different moral transgressions scenarios.

With respect to the selection of participants, most prior research relied on pre-selected samples from programs for gifted students without testing intelligence, while the current study draws on a sample for which intelligence was measured by an established cognitive ability test. More specific, a non-verbal intelligence test was used in order to measure inductive reasoning competencies independently from verbal abilities.

In summary, the current study wants to examine the impact of intelligence in the sense of inductive reasoning on children’s moral developmental status. In line with prior research, we assume that moral development is affected by intelligence. More specifically, we expect intelligence-related differences in reasoning about moral judgments. Furthermore, we expect a positive correlation between intelligence and moral motivation as well as between intelligence and negatively valenced moral emotions (NVMEs) which are both also indicators of moral development.

## Materials and Methods

### Participants

The sample size was determined based on the following considerations: statistical power of ≥0.90 and small to medium effect sizes of the expected correlations. A power analysis showed that considering a small to medium effect of *d* ≥ 0.30, at least 112 participants were necessary to achieve a power of ≥0.90 (G^∗^Power; Faul et al., 2007). So, we aimed at this figure as a minimum sample size. Participants were recruited from regular primary schools (*n* = 62) as well as from enrichment programs for gifted children (*n* = 67). Children had been assigned to gifted programs by teacher nomination. The aim of our recruiting approach was not to get two subsamples, we rather pursued a dual recruiting strategy in order to achieve an oversampling of highly intelligent children. Since most studies in this field examined participants from gifted programs, we were striving for an oversampling of gifted children. This procedure successfully resulted in two groups that only differ in their intelligence (see chap. Intelligence), but not in age or gender. However, although means of intelligence scores differ significantly between the two groups, there is a considerable overlap in the distribution of intelligence scores of both groups. A total of 129 children (*M* = 7 years; 7 months, *SD* = 7.5, range = 6 years; 4 months to 8 years; 10 months) from southwestern Germany participated in the study. The sample included more boys (*n* = 77) than girls (*n* = 52). Informed parental consent was obtained for all participants. The study with its procedural details was approved by the Ministry of Education, Youth and Sports, Baden-Württemberg.

### Procedure and Assessments

All children were interviewed individually by trained research assistants either in a separate room in their school or at their homes. Interview sessions lasted approximately 30–45 min. All interviews were audiotaped and later transcribed. Before the interview started, children were told that they were supposed to answer questions about picture stories. After a short warm-up task to familiarize children with the interview situation and the Likert-type smiley scale, all participants were presented four picture stories in randomized order. For all tasks, the names used in the stories matched the gender of the participant. After the interview, children were praised and rewarded with a little gift or candy. The intelligence test was conducted either in class as a group test or individually at the child’s home. The order of the two tasks (intelligence test and moral interview) was varied randomly.

#### Intelligence

Intelligence was assessed with subscales 3–5 of the CFT 1 by Weiß and Osterland (1997). The CFT1 is a partial adaption of the “Culture Fair Intelligence Tests – Scale 1” by Cattell (1950). It captures general intelligence with a main focus on general fluid ability defined by Cattell (1971), namely the ability to analyze cognitive problems in novel situations such as understanding rules or identifying patterns or relationships. Using only figural material, the test is non-verbal and it does not require any previous knowledge. Subtest 3 (classifications) focuses on identifying relations in figural problems with varying levels of difficulty. Subtest 4 (similarities) assesses the ability to compare and relate figural material. And subtest 5 (matrices) measures the ability to comprehend rules and relations in figural cognitive tasks. All three subtests have in common that they focus on relational thinking and the comprehension of rules and regularities which can be understood as a central part of general intelligence (Weiß and Osterland, 1997). IQ scores in the sample ranged from 82 to 145 (*M* = 117.6, *SD* = 11.4) with *M* = 122.3 (*SD* = 9.7) for the children from programs for the gifted, and *M* = 112.4 (*SD* = 11.0) for the children from regular primary schools.

It should be mentioned that the norms of the intelligence test that we used are from data mainly surveyed in the 1990s and thus slightly overestimate children’s IQ. We used that test, though, because it is an established instrument and has a good short version for research. This helped us keep the test sessions short which is important for this age group. Besides, for the purpose of our study, overage norms are not a problem because we did not want to make individual diagnostics. We need intelligence scores to relate them with moral development and we are interested in the covariation of the IQ scores with the indices of moral development, not in “absolute” intelligence scores. Further, despite the old norms, there were no ceiling effects in the IQ data and no significant deviation from normal distribution.

#### Moral Development

In order to assess children’s moral developmental status, four picture stories describing moral transgressions were used. More precisely, the stories were about not sharing with a needy child, stealing another child’s candy, hiding someone’s property, and picking on someone. This type of moral transgression stories has been frequently used and has been shown to be valid (Eisenberg, 1982; Nunner-Winkler and Sodian, 1988; Keller et al., 2003). Participants completed four measures for each story: (1) dichotomous act evaluation (“Is what the child did okay or not okay?”), (2) moral reasoning (responses to “Why?”), (3) Likert-type emotion attribution to self as victimizer (“What about you? If you had done that, how would you feel?” Likert-type: 1 = very bad to 4 = very good), and (4) justification for emotion attribution (responses to “Why?”). These four types of measures were used in all stories.

##### Coding and reliability

Participants’ justifications of act evaluations and attributed emotions were coded with four categories developed from the interviews themselves conforming with categories established in the literature (e.g., Malti et al., 2009a), including: (1) Moral reasons refer to moral norms or obligations (e.g., “it is not fair to get all the reward”) as well as to empathy or other’s welfare (e.g., “it’s wrong to do that because that would hurt him”). (2) Sanction-oriented reasons refer to an authority or sanctions by that authority (e.g., “you shouldn’t do that because if the teacher sees it, you will get into trouble”). (3) Hedonistic reasons refer to the satisfaction of personal needs and interests (e.g., “it’s ok to take it because I love candy”). (4) Undifferentiated reasons are unelaborated or uncodable (e.g., “Because he did it.” and “It’s not ok because it’s not ok.”).

All justifications were coded by the first author and approximately 25% of the interviews were coded by a second researcher for inter-rater reliability (Cohen’s *k* = 0.89). Justifications were coded as 1 = full use of the category; 0.5 = partial use with one other category; 0.33 = partial use with two other categories; 0 = no use of the category. This procedure was applied for proportional weighting of the use of multiple categories (thus proportions reflect the total sample). Analyses were conducted on proportional usage.

##### Moral development indices

In order to operationalize children’s moral development, two indices used in prior research were calculated: ‘Strength of Moral Motivation’ (e.g., Asendorpf and Nunner-Winkler, 1992; Malti et al., 2009b) and ‘NVMEs’ (Ongley and Malti, 2014). As the attributed emotions and the corresponding justifications are not independent (Nunner-Winkler, 2007), both indices combine attributed emotions with the corresponding reasoning. The attributed emotion in itself does not reliably tell us anything about the underlying motive and thus does not suffice to evaluate children’s moral developmental status. A combination of both – emotion and justification – helps us understand children’s (moral) motivation. In a first step, both types of indices were calculated separately for each story and in a second step, the scores were aggregated over all four stories resulting in two different sum scores of emotion attribution to self as victimizer and moral reasoning in the context of self-attributed emotions.

*Strength of moral motivation*. First, strength of moral motivation was calculated for each story separately. The scoring procedure follows the theoretical conceptualization of moral motivation as presented in the introduction. A score of 0 indicates attribution of a positive emotion justified by sanction-oriented, hedonistic or undifferentiated reasons. A score of 1 indicates attribution of a negative emotion justified by any reason that is not considered moral (hedonistic, sanction-oriented, undifferentiated). And a score of 2 indicates attribution of a negative emotion justified by moral reasons. Following Nunner-Winkler and Sodian (1988), a justification was considered moral as soon as a moral argument was mentioned. The mean scores for moral motivation range from 1.25 to 1.59 in the four stories [picking on someone: *M* = 1.25 (*SD* = 0.76); stealing: *M* = 1.59 (*SD* = 0.71); hiding someone’s property: *M* = 1.24 (*SD* = 0.84); not sharing: *M* = 1.40 (*SD* = 0.77)].

In a second step, the four moral motivation scores were aggregated over all stories (Cronbach’s α = 0.71). Only participants who got a moral motivation score for all four stories were included in the sum score (*N* = 125). Thus, the score had a range from 0 to 8 and a higher score indicates stronger moral motivation. The mean of moral motivation sum score was 5.54 (*SD* = 2.25, range: 0–8).

*Negatively valenced moral emotions (NVME)*. The concept of NVMEs or ‘guilt’ (e.g., Ongley and Malti, 2014; Colasante et al., 2016) also combines emotion attribution with the corresponding justifications differentiating between moral reasons versus all other types of reasons. A score of 0 = ‘no NVME’ indicates attribution of a positive emotion or a negative emotion justified by reasons which are not considered moral (hedonistic, sanction-oriented, undifferentiated). A score of 1 = ‘slight NVME’ indicates attribution of a slightly negative emotion (“rather bad”) with moral justifications and a score of 2 = ‘strong NMVE’ indicates a strong negative emotion (“very bad”) with moral justifications. This procedure allows for some gradation among the guilty or ’NVME’ responses acknowledging that children experience varying levels of NVMEs (Kochanska et al., 2002; Colasante et al., 2016). Again, justifications were considered moral as soon as any moral argument was mentioned. The mean scores for NVME range from 0.71 to 1.16 in the four stories [picking on someone: *M* = 0.71 (*SD* = 0.86); stealing: *M* = 1.16 (*SD* = 0.85); hiding someone’s property: *M* = 0.85 (*SD* = 0.91); not sharing: *M* = 0.87 (*SD* = 0.84)].

Analogous to the procedure concerning the moral motivation score, the four separate NVME scores were aggregated over all stories (Cronbach’s α = 0.64). Again, only participants who got a score for all four stories were included in the sum score (*N* = 125). A higher score indicates stronger NVMEs. The mean of NVME sum score was 3.66 (*SD* = 2.4, range: 0–8).

## Results

Repeated measures ANOVAs were used to test hypotheses related to differences in children’s reasoning about act evaluations by intelligence. Multiple regression analyses and rank correlations were used to test hypotheses related to the relationship between intelligence and moral developmental status.

### Act Evaluation

The act evaluation question was a control question in order to test if all children know the underlying moral rule and to exclude the possibility that the differences in emotion attribution are due to lack of moral knowledge. All participants (*N* = 129) evaluated all stories as wrong when completing the dichotomous act evaluation question. Therefore, all reasoning about act evaluation reference justifications why the act was wrong. See **Table 1** for an overview of children’s reasoning why the act is wrong.

**Table 1:** Proportions (standard deviations) of justifications for act evaluations and emotion attribution.

	Picking on someone	Stealing	Hiding someone’s property	Not sharing
**Act evaluation**				
Moral	0.77 (0.37)	0.89 (0.28)	0.85 (0.35)	0.81 (0.38)
Sanction-oriented	0.13 (0.27)	0.10 (0.25)	0.09 (0.27)	0.00 (0.00)
Hedonistic	0.03 (0.16)	0.00 (0.04)	0.02 (0.15)	0.06 (0.21)
Undifferentiated	0.07 (0.26)	0.02 (0.12)	0.04 (0.19)	0.13 (0.34)
**Emotion attribution**				
Moral	0.40 (0.47)	0.68 (0.44)	0.49 (0.49)	0.55 (0.47)
Sanction-oriented	0.17 (0.34)	0.13 (0.31)	0.16 (0.35)	0.01 (0.09)
Hedonistic	0.25 (0.41)	0.14 (0.33)	0.26 (0.43)	0.24 (0.40)
Undifferentiated	0.19 (0.39)	0.05 (0.21)	0.09 (0.28)	0.20 (0.40)

### Act Evaluation Reasoning and Intelligence

In a first step, correlation analyses were conducted in order to test whether there is a relation between intelligence and the use of moral reasoning. Analyses revealed no significant correlation coefficients between moral reasoning and intelligence in any of the stories (picking on someone: *r* = -0.12, *p* = 0.191; stealing: *r* = 0.10, *p* = 0.246; hiding someone’s property: *r* = 0.01, *p* = 0.918; not sharing: *r* = 0.02, *p* = 0.838).

In a second step, we followed the convention established by Turiel (2008) to use ANOVA-based procedures for reasoning analyses because they have been shown to be more appropriate for analyzing this type of data. In order to test the hypothesis that participants’ justifications for their act evaluation differ depending on their intelligence, a separate 2 (Gender: Male, Female) × 3 (Age: 6 year olds, 7 year olds, 8 year olds) × 3 (Justification: Moral, Sanction-oriented, Hedonistic) ANOVA with repeated measures on the last factor and intelligence as a covariate was conducted for each story. The repeated measures factor represents children’s reasoning about act evaluation. Means are proportions of use of the respective category. There was a significant main effect of justification in two of the stories [picking on someone: *F*(2,244) = 3.53, *p* = 0.031, η_p_^2^ = 0.03; hiding someone’s property: *F*(2,244) = 4.30, *p* = 0.015, η_p_^2^ = 0.03]. This revealed that participants for both stories used more moral justifications than any other reasons (*ps* < 0.001). Furthermore, for the not sharing story, there was a significant three-way interaction of justification, gender and age *F*(4,244) = 4.17, *p* = 0.003, η_p_^2^ = 0.06. In the group of 6 and 7 year olds, boys and girls displayed significant differences in the use of moral justifications (*ps* < 0.05). In the youngest age group, boys (*M* = 0.93, *SD* = 0.26) mentioned more moral reasons than girls (*M* = 0.69, *SD* = 0.46). The reverse finding emerged for the 7 year olds: girls (*M* = 0.95, *SD* = 0.22) mentioned more moral reasons than boys (*M* = 0.70, *SD* = 0.45). For the oldest age group, the 8 year olds, there were no differences between girls and boys.

However, for the current research question, the crucial results are those including intelligence as a covariate. More specifically, we expected intelligence-related differences in moral reasoning. Analyses revealed no significant main effects of intelligence in any of the stories [picking on someone: *F*(1,122) = 0.45, *p* = 0.505, η_p_^2^ = 0.00; stealing: *F*(1,122) = 1.33, *p* = 0.251, η_p_^2^ = 0.01; hiding someone’s property: *F*(1,122) = 0.02, *p* = 0.889, η_p_^2^ = 0.00; not sharing: *F*(1,122) = 2.94, *p* = 0.089, η_p_^2^ = 0.02]. Further, there was no significant interaction between intelligence and justifications. In other words, there were no intelligence-related differences in children’s reasoning about act evaluation.

### Emotion Attribution

Percentages of the attributed emotions for all stories can be found in **Table 2**. Analyses revealed no significant correlation coefficients between attributed emotions and intelligence in any of the stories (picking on someone: *r* = 0.16, *p* = 0.065; stealing: *r* = 0.09, *p* = 0.330; hiding someone’s property: *r* = 0.12, *p* = 0.174; not sharing: *r* = 0.16, *p* = 0.752). **Table 1** provides an overview of children’s justifications for their emotion attributions.

**Table 2:** Percentage of attributed emotions.

Attributed emotion	Picking on someone	Stealing	Hiding someone’s property	Not sharing
(1) Very bad	45	51.9	47.7	42.1
(2) Rather bad	35.7	35.7	26.6	40.5
(3) Rather good	10.9	7.8	11.7	13.5
(4) Very good	8.5	4.7	14.1	4.0

### Moral Development Indices and Intelligence

The attributed emotion in itself does not suffice to evaluate children’s moral developmental status because it does not reliably tell us anything about the underlying motive. Thus, in the following analyses moral development indices are used which combine both – emotion attribution and justification.

In order to further test the impact of intelligence on children’s moral development, multiple regression analyses on moral motivation and on NVMEs were conducted for all stories separately, including intelligence, age and gender as predictors. Further, to test the hypothesis that participants’ moral development is positively related to their intelligence, rank correlations between moral motivation and intelligence as well as between NVMEs and intelligence were calculated.

Multiple regression analyses showed that intelligence was not a significant predictor for moral motivation or NVMEs in any of the stories (see **Table 3** for *t*-values of regression coefficients). Analyses with the aggregated scores did not show intelligence to be a significant predictor, either [moral motivation: *t*(121) = 0.58, *p* = 0.563; NVME: *t*(121) = -0.41, *p* = 0.683]. In the story picking on someone age contributed significantly to moral motivation [*t*(125) = -2.17, *p* = 0.032]. In the story hiding someone’s property gender revealed as a significant predictor of moral motivation [*t*(124) = -2.33, *p* = 0.032]. For the aggregated score moral motivation score, age and gender significantly contributed to the variance [age: *t*(121) = -2.33, *p* = 0.021; gender: *t*(121) = -2.51, *p* = 0.013]. Since the results on age and gender are not relevant for the current research question, they are not further considered.

**Table 3:** *t*-values of regression coefficients with *p*-values in parentheses.

	Picking on someone	Stealing	Hiding someone’s property	Not sharing
Moral motivation	*t*(125) = 0.72 (*p* = 0.475)	*t*(125) = 0.00 (*p* = 0.998)	*t*(124) = 0.23 (*p* = 0.820)	*t*(122) = 0.99 (*p* = 0.325)
NVME	*t*(125) = 0.89 (*p* = 0.374)	*t*(125) = -1.53 (*p* = 0.128)	*t*(124) = -0.01 (*p* = 0.993)	*t*(122) = -0.52 (*p* = 0.605)

Correlation analyses revealed no significant correlation coefficients between moral development and intelligence in any of the stories. We found no significant correlations with intelligence for either moral motivation or for NVMEs, see **Table 4**. Correlation analyses with the aggregated scores did not reveal any significant results, either (moral motivation: *r* = 0.04, *p* = 0.632; NVME: *r* = -0.03, *p* = 0.713).

**Table 4:** Correlations of moral scores and intelligence.

	Picking on someone	Stealing	Hiding someone’s property	Not sharing
Moral motivation	*r* = 0.08 (*p* = 0.348)	*r* = -0.04 (*p* = 0.647)	*r* = 0.01 (*p* = 0.938)	*r* = 0.07 (*p* = 0.436)
NVME	*r* = 0.09 (*p* = 0.314)	*r* = -0.15 (*p* = 0.091)	*r* = -0.02 *(p* = 0.825)	*r* = -0.02 (*p* = 0.804)
*N*	129	129	128	126

## Discussion

The purpose of the current study was to investigate whether intelligence affects moral development as assessed across a range of different moral transgressions. Thereby, the study was designed to address some of the shortcomings of prior research by examining younger children and applying an approach that is more closely connected to children’s daily lives. By including moral emotions, a broader conceptualization of moral development was chosen than in prior research in the field which has often been restricted to moral judgments or moral reasoning about those judgments. Furthermore, we did not rely on a pre-selected group of gifted children, but directly measured children’s intelligence. Altogether, 129 children aged between 6 years; 4 months and 8 years; 10 months were interviewed using four different moral transgression stories.

Given our results, findings from prior research with adolescents or adults cannot simply be extended to younger participants. We found no significant correlations between moral development and intelligence in any of the stories. Neither for moral cognitions, nor for moral emotions, did we find any evidence for intelligence-related differences. More specifically, there were no significant correlations between moral motivation and intelligence and no correlations between NVMEs and intelligence, either. Neither did we find any intelligence-related differences in moral reasoning about act evaluations. Therefore, our findings indicate that for children aged between 6 years; 4 months and 8 years; 10 months, inductive reasoning competencies, i.e., intelligence, cannot explain differences in moral development.

At a first glance, this seems to contradict prior findings which demonstrated strong connections between intelligence and morality. How did prior research differ from ours, and why do we expect our study to be more apt than prior ones?

Firstly, as we have illustrated in the introduction, research that examined the association of morality and intelligence, typically used a different approach to measure morality. Our concern is that traditional measures like the DIT or the Moral Judgment Interview are quite disconnected to everyday life and the results of these instruments rather represent an ability to evaluate different moral principles in a prescriptive way, i.e., what *should* be done in the respective situation (Elm and Weber, 1994). Thus, it is questionable if they really measure a person’s morality, i.e., a person’s level of moral development, in the sense of what a person would really decide to do in morally relevant situations. It could be assumed that this ability – which can rather be considered as philosophizing about moral problems – is stronger connected to intellectual abilities than the attribution of emotions or the reasoning about act evaluations in the type of moral transgression stories we used in the current study. Especially, as the dilemmas used in the DIT or in the Moral Judgment Interview are very complex and abstract, and remote from the kind of moral problems faced by people in their everyday lives. Correspondingly, Malti et al. (2013) concluded based on their findings that it is reasonable to assume a connection between intelligence and moral reasoning but not between intelligence and moral emotions.

Some authors go even further and claim that the DIT is not conceptually distinct from measures of verbal ability, meaning that DIT scores are reducible to intellectual ability (Sanders et al., 1995). In other words, it is even questionable if prior results really show a connection between morality and intelligence or if they can be considered an artifact that is produced because the instrument is confounded with intelligence.

Furthermore, many prior studies relied on pre-selected groups of gifted children (e.g., from summer camps or other enrichment programs). It is often unclear which criteria the classification of giftedness is based on in these programs. Usually, children are nominated by teachers, and teacher judgments are biased by the children’s families’ socio economic background and personality traits like conscientiousness or effort, etc. (Baudson, 2010). Thus, it is unclear whether the findings are really due to intelligence or whether they might be caused by other factors which are typical for some gifted children. In the current study, we did not rely on such a pre-selection. We recruited children from regular elementary schools as well as from a program for gifted children, but we directly measured intelligence in all children who participated and found no relationship between intelligence and our measures of morality. This aligns with research from the field of prosocial development which is closely related to moral development. For example Paulus et al. (2015) assessed intelligence and working memory and did not find any significant correlations between general cognitive abilities and sharing behavior, either.

However, some points remain to be mentioned and discussed. First of all, we interviewed children at elementary school age. Needless to say, we cannot rule out the possibility that intelligence does matter in even younger children. It is conceivable that in younger age groups, intelligence leads to differences in moral development and in our sample the influence of intelligence just does not come into effect due to a threshold effect, meaning that above a certain degree of intelligence there might be no relation to moral development. In terms of information processing, this could mean that a certain degree of intelligence is necessary to adequately process the extent of information that is at play in morally relevant situations. Accordingly, it is plausible to assume that for the normal range of intelligence as well as for the higher range, there is no relation between intelligence and moral development. Thus, to address this question, future studies should include even younger children and children with lower intelligence scores.

Secondly, further research needs to replicate the current findings with other samples and multiple measures. For example, it is conceivable that moral reasoning ability is more related to verbal intelligence than to general intelligence measured with a non-verbal instrument. However, the aim of the current study was to examine the relationship between moral development and inductive reasoning independently of verbal abilities.

Further, it was surprising that we did not find any age-related differences for the emotion attribution questions and for the moral motivation sum score, and only a small but significant correlation with the NVME sum score. So, one might think that the children were too old and did not differ in their answers at all. However, there were no ceiling effects in any of the moral variables and the answers showed a lot of variation, albeit without relation to the children’s age. One possible explanation for the lack of age-related findings might be that in the current study, children’s age range is limited to 2.5 years in contrast to other studies including wider ranges.

Moreover, some researchers might question the procedure of considering a justification to be moral as soon as one moral argument is mentioned. Some authors argue that it is better to use the first, spontaneous, answer (Ongley and Malti, 2014). Accordingly, a justification is only considered moral if the first answer is a moral one. But we assume that the spontaneous answer is not necessarily the most important argument for the child, because the order of reasons that children provide can also be influenced by other things, e.g., situational aspects (Nunner-Winkler and Sodian, 1988). Thus, we argue that when a child is able to express a moral argument, this argument does have certain significance for the child and therefore can be understood in the sense of moral motivation.

All in all, the findings of the current study provide some first evidence that moral development – measured in a way that is closely connected to everyday life and across a range of different moral transgression scenarios – is not affected by children’s general intelligence in the sense of inductive reasoning measured with figural material. Of course, it cannot be denied, that children need a certain amount of cognitive abilities in morally relevant situations to coordinate perspectives, select and process relevant information, anticipate consequences, and interpret the whole situation (Dentici and Pagnin, 1992; Derryberry et al., 2005). But given our findings, it can be assumed that young children already have a sufficient minimum level of cognitive abilities to successfully manage morally relevant situations. Thus, individual differences in children’s moral development need to be explained by other factors than intelligence. Nonetheless, the current findings still need to be replicated in further studies.

## Author Contributions

Both authors contributed substantially to the conception and design of the study as well as to the planning of the analyses and interpretation of data. HB drafted the manuscript, and it was critically revised by MH. Both authors approve the final version to be published, and agree to be accountable for all aspects of the work in ensuring that questions related to the accuracy or integrity of any part of the work are appropriately investigated and resolved.

## Conflict of Interest Statement

The authors declare that the research was conducted in the absence of any commercial or financial relationships that could be construed as a potential conflict of interest.

## References

[B1] Asendorpf J. B. Nunner-Winkler G. (1992). Children’s moral motive strength and temperamental inhibition reduce their immoral behavior in real moral conflicts. *Child Dev.* 63 1223–1235. 10.1111/j.1467-8624.1992.tb01691.x

[B2] Baudson T. G. (2010). “Nominationen von Schülerinnen und Schülern für Begabtenfördermaßnahmen. [Nomination of students for programs for the gifted],” in *Diagnostik von Hochbegabung [Diagnostics of giftedness]* Vol. 8 eds Preckel F. Schneider W. Holling H. (Göttingen: Hogrefe Verlag) 383–431.

[B3] Cattell R. B. (1950). *Culture Fair Intelligence Test: A Measure of ”g”*. Savory, IL: Institute for Personality and Ability Testing.

[B4] Cattell R. B. (1971). *Abilities: Their Structure, Growth, and Action*. New York, NY: Houghton Miﬄin.

[B5] Chovan W. Freeman N. L. (1993). Moral reasoning and personality components in gifted and average students. *Percept. Mot. Skills* 77 1297–1298. 10.2466/pms.1993.77.3f.1297 8170784

[B6] Colasante T. Zuffianò A. Malti T. (2016). Daily deviations in anger, guilt, and sympathy: a developmental diary study of aggression. *J. Abnorm. Child Psychol.* 44 1515–1526. 10.1007/s10802-016-0143-y 27000823

[B7] Colby A. Kohlberg L. (1987). *The Measurement of Moral Judgment: Theoretical Foundations and Research Validations*. Cambridge, MA: Cambridge University Press.

[B8] Dentici O. A. Pagnin A. (1992). Moral reasoning in gifted adolescents: cognitive level and social values. *Eur. J. High Abil.* 3 105–114. 10.1080/0937445920030111

[B9] Derryberry W. P. Wilson T. Snyder H. Norman T. Barger B. (2005). Moral judgment developmental differences between gifted youth and college students. *Prufrock J.* 17 6–19. 10.4219/jsge-2005-392

[B10] Eisenberg N. (1982). *The Development of Prosocial Behavior.* New York, NY: Academic Press.

[B11] Eisenberg N. (2000). Emotion, regulation, and moral development. *Annu. Rev. Psychol.* 51 665–697. 10.1146/annurev.psych.51.1.665 10751984

[B12] Eisenberg N. Lennon R. Roth K. (1983). Prosocial development: a longitudinal study. *Dev. Psychol.* 19 846–855. 10.1037/0012-1649.19.6.846

[B13] Elm D. R. Weber J. (1994). Measuring moral judgment: the moral judgment interview or the defining issues test? *J. Bus. Ethics* 13 341–355. 10.1007/BF00871762

[B14] Faul F. Erdfelder E. Lang A. G. Buchner A. (2007). G^∗^ Power 3: a flexible statistical power analysis program for the social, behavioral, and biomedical sciences. *Behav. Res. Methods* 39 175–191. 10.3758/BF03193146 17695343

[B15] Gibbs J. C. (2013). *Moral Development and Reality: Beyond the Theories of Kohlberg, Hoffman, and Haidt*. Oxford: Oxford University Press.

[B16] Gross M. U. (1993). *Exceptionally Gifted Children.* Abingdon: Routledge.

[B17] Hoffman M. (2000). *Empathy and Moral Development: The Implications for Caring and Justice*. Cambridge: Cambridge University Press.

[B18] Hoffman M. L. (1991). “Empathy, social cognition, and moral action,” in *Handbook of Moral Behavior and Development* Vol. 1 eds Kurtines W. L. Gewirtz J. L. (Hillsdale, NJ: Erlbaum) 275–301.

[B19] Howard-Hamilton M. (1994). An assessment of moral development in gifted adolescents. *Roeper Rev.* 17 57–59. 10.1080/02783199409553621

[B20] Howard-Hamilton M. Franks B. A. (1995). Gifted adolescents: psychological behaviors, values, and developmental implications. *Roeper Rev.* 17 186–191. 10.1080/02783199509553656

[B21] Janos P. M. Robinson N. M. (1985). “Psychosocial development in intellectually gifted children,” in *The Gifted and Talented: Developmental Perspective* eds Horowitz F. D. O’Brien M. (Washington, DC: American Psychological Association) 149–195.

[B22] Kail R. Salthouse T. A. (1994). Processing speed as a mental capacity. *Acta Psychol.* 86 199–225. 10.1016/0001-6918(94)90003-5 7976467

[B23] Karnes F. A. Brown K. E. (1981). Moral development and the gifted: an initial investigation. *Roeper Rev.* 3 8–10. 10.1080/02783198109552540

[B24] Keller M. (2001). “Moral in Beziehungen: Die Entwicklung des frühen moralischen Denkens in Kindheit und Jugend [morality in relationships: development of early moral thinking in childhood and adolescents],” in *Moralische Erziehung in der Schule: Entwicklungspsychologie und pädagogische Praxis.[Moral Education at School: Developmental Psychology and Educational Practice* eds Edelstein W. Oser F. Schuster P. (Weinheim: Beltz) 111–140.

[B25] Keller M. Lourenço O. Malti T. Saalbach H. (2003). The multifaceted phenomenon of ‘happy victimizers’: a cross-cultural comparison of moral emotions. *Br. J. Dev. Psychol.* 21 1–18. 10.1348/026151003321164582

[B26] Kochanska G. Gross J. N. Lin M. H. Nichols K. E. (2002). Guilt in young children: development, determinants, and relations with a broader system of standards. *Child Dev.* 73 461–482. 10.1111/1467-8624.00418 11949903

[B27] Kohlberg L. (1964). “Development of moral character and moral ideology,” in *Review of Child Development Research* eds Hoffman M. Hoffman L. (New York, NY: Russell Sage Foundation) 383–431.

[B28] Kohlberg L. (1969). “Stage and sequence: the cognitive-developmental approach to socialization,” in *Handbook of Socialization: Theory and Research* ed. Goslin D. A. (Chicago: Rand McNally) 347–380.

[B29] Kohlberg L. (1975). The cognitive-developmental approach to moral education. *Phi Delta Kappan* 56 670–677.

[B30] Lee S. Y. Olszewski-Kubilius P. (2006). The emotional intelligence, moral judgment, and leadership of academically gifted adolescents. *J. Educ. Gifted* 30 29–67. 10.1177/016235320603000103

[B31] Malti T. Eisenberg N. Kim H. Buchmann M. (2013). Developmental trajectories of sympathy, moral emotion attributions, and moral reasoning: the role of parental support. *Soc. Dev.* 22 773–793.

[B32] Malti T. Gasser L. Buchmann M. (2009a). Aggressive and prosocial children’s emotion attributions and moral reasoning. *Aggress. Behav.* 35 90–102. 10.1002/ab.20289 18985747

[B33] Malti T. Gummerum M. Keller M. Buchmann M. (2009b). Children’s moral motivation, sympathy, and prosocial behavior. *Child Dev.* 80 442–460. 10.1111/j.1467-8624.2009.01271.x 19467003

[B34] Malti T. Krettenauer T. (2013). The relation of moral emotion attributions to prosocial and antisocial behavior: a meta-analysis. *Child Dev.* 84 397–412. 10.1111/j.1467-8624.2012.01851.x 23005580

[B35] Malti T. Latzko B. (2010). Children’s moral emotions and moral cognition: towards an integrative perspective. *New Dir. Child Adolesc. Dev.* 129 1–10. 10.1002/cd.272 20872601

[B36] Nunner-Winkler G. (1998). “Zum Verständnis von Moral – Entwicklungen in der Kindheit [understanding morality: development in childhood],” in *Entwicklung im Kindesalter [Development in Childhood]* ed. Weinert F. E. (Weinheim: Psychologische Verlags Union) 133–152.

[B37] Nunner-Winkler G. (2007). Development of moral motivation from childhood to early adulthood. *J. Moral Educ.* 36 399–414. 10.1080/03057240701687970

[B38] Nunner-Winkler G. (2009). “Moral motivation from childhood to early adulthood,” in *Human Development from Early Childhood to Early Adulthood: Findings from a 20 year Longitudinal Study* eds Schneider W. Bullock M. (New York, NY: Psychology Press) 91–118.

[B39] Nunner-Winkler G. Sodian B. (1988). Children’s understanding of moral emotions. *Child Dev.* 50 1323–1338. 10.2307/1130495 3168643

[B40] Ongley S. F. Malti T. (2014). The role of moral emotions in the development of children’s sharing behavior. *Dev. Psychol.* 50 1148–1159. 10.1037/a0035191 24294881

[B41] Paulus M. Licata M. Kristen S. Thoermer C. Woodward A. Sodian B. (2015). Social understanding and self-regulation predict pre-schoolers’ sharing with friends and disliked peers A longitudinal study. *Int. J. Behav. Dev.* 39 53–64. 10.1177/0165025414537923

[B42] Rest J. R. (1979). *Developing in Judging Moral Issues.* Minneapolis, MN: University of Minnesota.

[B43] Rest J. R. (1986). *Moral Development: Advances in Research and Theory*. New York, NY: Praeger publishers.

[B44] Rest J. R. Narvaez D. Bebeau M. Thoma S. (1999). A neo-Kohlbergian approach: the DIT and schema theory. *Educ. Psychol. Rev.* 11 291–324. 10.1023/A:1022053215271

[B45] Rost D. H. Czeschlik T. (1994). The psycho-social adjustment of gifted children in middle-childhood. *Eur. J. Psychol. Educ.* 9 15–25. 10.1007/BF03172882

[B46] Rost D. H. Hanses P. (1997). Wer nichts leistet, ist nicht begabt? Zur Identifikation hochbegabter Underachiever durch Lehrkräfte. [Those not achieving are not gifted? Teachers‘ identifications of gifted underachievers]. *Z. Entwicklungspsychol. Pädagog. Psychol.* 29 167–177.

[B47] Sanders C. E. Lubinski D. Benbow C. P. (1995). Does the defining issues test measure psychological phenomena distinct from verbal ability? An examination of Lykken’s query. *J. Pers. Soc. Psychol.* 69 498–504. 10.1037/0022-3514.69.3.498

[B48] Silverman L. K. (1994). The moral sensitivity of gifted children and the evolution of society. *Roeper Rev.* 17 110–116. 10.1080/02783199409553636

[B49] Smetana J. G. (2006). “Social-cognitive domain theory: consistencies and variations in children’s moral and social judgments,” in *Handbook of Moral Development* eds Killen M. Smetana J. (Mahwah, NJ: Erlbaum) 119–153.

[B50] Tan-Willman C. Gutteridge D. (1981). Creative thinking and moral reasoning of academically gifted secondary school adolescents. *Gifted Child Q.* 25 149–154. 10.1177/001698628102500402

[B51] Thorndike R. L. (1940). Performance of gifted children on tests of developmental age. *J. Psychol.* 9 337–343. 10.1080/00223980.1940.9917700

[B52] Turiel E. (1983). *The Development of Social Knowledge: Morality and Convention*. Cambridge, MA: Cambridge University Press.

[B53] Turiel E. (2008). Thought about actions in social domains: morality, social conventions, and social interactions. *Cogn. Dev.* 23 136–154. 10.1016/j.cogdev.2007.04.001

[B54] Weiß R. H. Osterland J. (1997). *Grundintelligenztest Skala 1 (CFT 1). 5., revidierte Auflage [Culture Fair Intelligence Test – Scale 1]* Vol. 5. Göttingen: Hogrefe.

